# Measuring Medical Students' Preparedness and Skills to Provide Cross-Cultural Care

**DOI:** 10.1089/heq.2016.0011

**Published:** 2017-01-01

**Authors:** Alexander R. Green, Maria B.J. Chun, Marina C. Cervantes, Jacob D. Nudel, Jason V. Duong, Edward Krupat, Joseph R. Betancourt

**Affiliations:** ^1^ Division of General Internal Medicine, Massachusetts General Hospital and Harvard Medical School, Boston, Massachusetts.; ^2^ Department of Surgery, John A. Burns School of Medicine, University of Hawaii at Manoa, Honolulu, Hawaii.; ^3^ Department of Family and Community Medicine, University of California-San Francisco, San Francisco, California.; ^4^ Department of Surgery, Boston University School of Medicine, Boston, Massachusetts.; ^5^ Yale School of Public Health, New Haven, Connecticut.; ^6^ Center for Evaluation, Harvard Medical School, Boston, Massachusetts.; ^7^ Disparities Solutions Center, Massachusetts General Hospital and Harvard Medical School, Boston, Massachusetts.

**Keywords:** cross-cultural care, cultural competency, medical education

## Abstract

**Purpose:** Cross-cultural education is an integral and required part of undergraduate medical curricula. However, the teaching of cross-cultural care varies widely and methods of evaluation are lacking. We sought to better understand medical students' perspectives on their own cultural competency across the 4-year curriculum using a validated survey instrument.

**Methods:**We conducted an annual Internet-based survey at Harvard Medical School with students in all 4 years of training, for four consecutive years. We used a tool previously validated with residents and slightly modified it for medical students, assessing their (1) preparedness, (2) skillfulness, and (3) perspectives on the educational curriculum and learning climate.

**Results:** Of 2592 possible survey responses, we received 1561 (60% response rate). Fourth-year students had significantly higher scores than first-year students (*p*<0.001) for all but one preparedness item (caring for transgender patients) and all but one skillfulness item (identifying ability to read/write English). Less than 50% of students felt adequately prepared/skilled by their fourth year on 8 of 11 preparedness items and 5 of 10 skillfulness items. Lack of practical experience caring for diverse patients was the most frequently cited challenge.

**Conclusions:** While students reported that preparedness and skillfulness to care for culturally diverse patients seem to increase with training, fourth-year students still felt inadequately prepared and skilled in many important aspects of cross-cultural care. Medical schools can use this tool with students to self-assess cultural competency and to help guide enhancements to their curricula focusing on cross-cultural care.

## Introduction

Over the past few decades, medicine has undergone a transition from a paternalistic, “one-size-fits-all” approach, to a more patient-centered perspective.^1,2^ Research has shown that patient-centered care improves quality of care, patient satisfaction, health outcomes, and quality of life, and decreases healthcare utilization.^3^ With the rich cultural diversity of the United States, tailoring care to meet patients' unique perspectives is challenging and has driven the teaching of cross-cultural care in health professions curricula. Accrediting bodies, such as the Liaison Committee on Medical Education (LCME) and the Accreditation Council for Graduate Medical Education (ACGME), include cultural competency as part of their standards for training medical students and residents.^4,5^ However, physicians-in-training do not feel adequately prepared to provide patient-centered care in a cross-cultural context.^6^ In a national survey, 25% of senior residents reported feeling unprepared to provide care to new immigrants and to patients with health beliefs at odds with Western medicine, while 20% felt unprepared to care for patients whose religious beliefs affect treatment.^6^ In another study, 80% of medical students did not feel well prepared to care for patients with limited English proficiency (LEP).^7^

Although many medical schools include cross-cultural education within required courses,^8^ there is great variability in content and teaching approaches.^9–12^ For example, some schools focus more on cultural information and less on practical skills, and many underemphasize bias, stereotyping, and disparities. There is also variability in the timing of training,^13^ with many medical schools emphasizing cultural competency and cross-cultural care in the first or second year, without integration across the 4-year continuum.^14,15^ Given the lack of standardization, evaluating the impact of curricular innovations is challenging.^10,16,17^ Also, the “hidden curriculum”—the informal lessons learned by students through interactions with faculty, residents, patients and staff—strongly influences students' attitudes and perspectives,^18,19^ and likely plays a role in how students learn about cross-cultural care.

At Harvard Medical School (HMS), cross-cultural care is not taught as a specific course, but is woven throughout the curriculum through various experiences, led whenever possible by faculty with expertise in this area. The Cross-Cultural Care Committee at HMS has promoted this process for over a decade, integrating these issues into various courses wherever possible. As part of a new curriculum reform process in 2015, Cross-Cultural Care and Health Equity has now been designated as one of the key themes to be integrated into every course across the 4-year continuum. This integrative approach ideally allows students to learn the concepts and skills of cross-cultural care in the context of patient-centered medical care more generally rather than as a separate issue for specific patient groups. However, it is difficult to standardize the educational experience students receive and to evaluate the impact on students' learning.

Tools to assess cultural competence education at the medical undergraduate level exist. For example, the Association of American Medical Colleges Tool for Assessing Cultural Competence Training (TACCT) allows medical schools to review how cultural competency has been implemented in the curriculum.^20^ The Health Beliefs Attitudes Survey (HBAS) and the Sociocultural Attitudes in Medicine Inventory (SAMI) assess students' attitudes toward cross-cultural care. However, these scales do not measure knowledge, skills, or perceived preparedness.^21,22^ In 2005, we developed the Cross-Cultural Care Survey (CCCS) to assess self-perceived skills and preparedness to deliver cross-cultural care among resident physicians, and to assess the cultural climate and learning environment at their institutions.^6^ The CCCS is one of the few tools focused on cultural competency in medicine that has been formally validated.^23,24^

To gain a deeper understanding of medical students' perspectives on cross-cultural care and education at HMS as well as their level of cultural competency in terms of both preparedness and skills across the 4-year continuum, we implemented a longitudinal, annual survey at each class level at the beginning of each year. We used a slightly modified version of the CCCS that included some questions specific to the HMS curriculum. We did not implement a specific intervention as part of this study; rather, we used the CCCS to assess the existing curriculum. We hypothesized, among other things, that fourth-year students would have greater preparedness and skills than first-year students. We report data from four survey years, focusing primarily on preparedness, skillfulness, and the learning climate.

## Methods

### Survey design and administration

We designed an Internet-based survey for medical students to explore their preparedness and skillfulness at providing cross-cultural care. We also asked about the learning environment and basic personal and professional characteristics. We used the CCCS with some minor modifications.^6^ We added three items to explore students' preparedness to care for patients who are (1) gay, lesbian, or bisexual, (2) transgender, and (3) persons with disabilities. We slightly modified the skillfulness scale by removing references to “pediatric patient's family” and adding an item on counseling patients on their use of alternative or complementary medicine. We also added some new questions on attitudes that we did not include in the analyses for this study. Our study protocol was deemed exempt by the Institutional Review Boards of both HMS and the Partners HealthCare System.

We administered the survey electronically from mid-August to mid-October in 2009, 2010, 2011, and 2012 to all medical students, years 1–4. This corresponds to the beginning of the academic year, when first-year students still had minimal exposure to the formal and informal educational curriculum. We excluded dental and MD/PhD students from the study due to differences in training schedule and curriculum. Participation in the online survey was voluntary for all students.

To enhance our response rate, we sent four reminder e-mails to students separated by 5 to 10 days. We also entered participants into a random prize drawing for gift cards. Eight students were awarded gift cards each survey year (three $500 gift cards and five $200 gift cards). Completion and return of the survey constituted consent.

### Variables

We measured two constructs related to cross-cultural care: (1) preparedness to care for specific types of patients and (2) self-assessment of specific cross-cultural skills. We also included questions assessing the educational curriculum and climate around cross-cultural care.

#### Preparedness

To assess their preparedness, we asked medical students how prepared they felt to care for a series of types of patients (1=*Very Unprepared*, 2=*Somewhat Unprepared*, 3=*Somewhat Prepared*, 4=*Well Prepared*, 5=*Very Well Prepared*). The list included patients from cultures different from their own, patients with health beliefs at odds with Western medicine, patients with religious beliefs that might affect treatment, patients who were new immigrants, patients with LEP, lesbian, gay, bisexual, and transgender patients, patients with disabilities, and patients who use alternative or complementary medicines. Responses of *Very Unprepared*, *Somewhat Unprepared*, and *Somewhat Prepared* were combined to indicate lack of preparedness and responses of *Well Prepared* and *Very Well Prepared* were combined to indicate preparedness.

#### Skillfulness

Students also were asked to assess their skill level in performing certain functions thought to be useful in treating culturally diverse patients, or pediatric patients' families (1=*Not at All Skillful* to 5=*Very Skillful*). These included determining how to address patients from different cultures, assessing patients' understanding of their illness, identifying mistrust, negotiating treatment plans, assessing English proficiency, identifying relevant cultural and religious beliefs, understanding decision-making roles, working with interpreters, and counseling patients about their use of complementary or alternative medicine.

#### Training and educational climate

Other key variables include questions on the educational curriculum and an assessment of the educational climate. To assess the curriculum, students were asked to specify the courses completed and how each class prepared them to interact with culturally diverse patients using the following rating scale: (1=*Not at All Useful*, 2=*Somewhat Useful*, 3=*Useful*, 4=*Very Useful*, and 5=N/A). To assess the educational climate, we asked medical students to rate “how much of a problem” (1=*No Problem*, 2=*Small Problem*, 3=*Moderate Problem*, and 4=*Big Problem*) the following had been during medical school: (1) lack of practical experience caring for diverse patients; (2) inadequate cross-cultural care training; (3) absence of good role models or mentors in cross-cultural care; (4) dismissive attitudes about cross-cultural care among physicians; and (5) dismissive attitudes about cross-cultural care among fellow students. We also asked students to rate (1=*Strongly Disagree*, 2=*Somewhat Disagree*, 3=*Slightly Disagree*, 4=*Slightly Agree*, 5=*Somewhat Agree*, 6=*Strongly Agree*) their level of agreement on the inclusion of cross-cultural care curricula into courses and clinical practice.

Other questions assessed medical student demographic characteristics, including gender, race/ethnicity, language, and school year.

### Analysis

Chi-square analyses were used to test for differences in preparedness and skillfulness items between years within the curriculum. All preparedness and skillfulness item responses were dichotomized into “Unprepared/Unskilled” (response of “1,” “2,” or “3”) and “Prepared/Skilled” (response of “4” or “5”) and examined for change between year 1 and 4. In addition, the item responses were categorized into the three levels of “Unprepared/Unskilled” (response of “1” or “2”), “Intermediate” (response of “3”), and “Prepared/Skilled” (response of “4” or “5”), and tested for change across all years in the curriculum. Furthermore, responses about the extent of problems during medical training were dichotomized into the categories of “No Problem” (response of “1”) and “Problem” (responses of “2,” “3,” and “4”) and analyzed with Crosstab analyses. A chi-square also was used to investigate differences in the proportion of white students between years to provide possible explanations for differences in the other variables between years. Because of multiple tests, a Bonferroni correction was made to the alpha level to control for family-wise error rate, generating a significance criteria of *p*<0.002. Demographics were examined using frequency analyses. All analyses were conducted using SPSS version 20.

## Results

Of 2592 possible survey responses across the 4 years of the study, we received 1561 responses. Of those, we excluded 192 due to incomplete responses (143), invalid responses (7), and duplicates (42). After accounting for these exclusions, the final sample was 1369 (response rate 60.2%). Of note, students were encouraged to repeat the survey in subsequent years, so these results represent fewer than 1369 individual students. All results reported were also analyzed separately for the four distinct survey years and compiling the data did not significantly change any of the main results.

### Respondent characteristics

Table 1 shows some basic demographic characteristics for the medical students who participated in this study. All 4 years were similar. With regard to gender, there were slightly more females who participated than males, for all but third-year students (male=51.7%; female=48.3%). For racial/ethnic minorities, the distribution was similar across the 4 years. The average number of white students was 46.4%, with the second largest group comprising Asians/Pacific Islanders (30.9%). The percentage of students for whom English was their primary language was also similar across the 4 years (73.9%).

**Table 1.** **Demographics of Student Respondents, Harvard Medical School, 2009–2012**

	% of respondents (*N*=1369)				
	Year 1	Year 2	Year 3	Year 4	Year 1–4
Gender					
Male	49.4	46.3	51.7	45.2	48.1
Female	50.6	53.7	48.3	54.5	51.9
Other	0.0	0.0	0.0	0.3	0.1
Total	100.0	100.0	100.0	100.0	100.0
Race/ethnicity					
White	43.2	48.5	46.3	47.8	46.4
Black	7.5	6.2	7.3	9.1	7.5
Asian or Pacific Islander	33.8	32.2	32.4	25.6	30.9
Native American/Alaskan Native	0.2	0.3	1.0	1.0	0.6
Hispanic/Latino/a	8.5	8.6	6.0	5.1	7.1
Other	6.8	4.3	7.0	11.4	7.4
Total	100.0	100.0	100.0	100.0	100.0
First language					
English	72.1	72.0	74.3	77.5	73.9
Other	27.9	28.0	25.7	22.5	26.1
Total	100.0	100.0	100.0	100.0	100.0

2.4%, 3.6%, 3.2% missing in each respective category.

### Preparedness and skillfulness

Table 2 shows 11 measures of students' self-reported preparedness and 10 measures of self-reported skillfulness specific to cross-cultural care. We ordered the findings based on the absolute percentage difference in reports of adequate preparedness or skillfulness between students entering medical school and those entering the fourth year. For the 11 preparedness items, the mean absolute percent difference between beginning fourth-year students and beginning first-year students was 16.7% with a range from 7.3% to 30.5%. For the 10 skills items, the mean difference was 33.1% with a range from 5.2% to 60.5%.

**Table 2.** **Differences in First- and Fourth-Year Medical Students' Preparedness and Skillfulness to Provide Cross-Cultural Care, Harvard Medical School, 2009–2012**

	% Difference (*N*=1369)				
	Year 1	Year 2	Year 3	Year 4	Between year 1–4
With limited English proficiency^*^	11.6	18.0	30.8	42.2	30.6
From cultures different from your own^*^	27.7	34.1	41.5	54.6	26.9
Who are members of racial/ethnic minorities^*^	50.9	59.1	66.2	73.2	22.3
Who are persons with disabilities^*^	23.8	21.1	28.0	41.0	17.2
Who use complementary or alternative medicines^*^	11.8	21.7	25.5	26.7	14.9
Who are gay, lesbian, or bisexual^*^	48.4	48.0	58.4	63.1	14.7
With a distrust of the U.S. healthcare system^*^	10.9	14.3	17.4	24.4	13.5
With health beliefs or practices at odds with Western medicine^*^	10.2	12.2	16.5	22.9	12.7
Whose religious beliefs affect treatment^*^	10.5	15.6	21.1	23.0	12.5
Who are new immigrants^*^	18.0	20.4	23.9	29.9	11.9
Who are transgender^**^	18.3	19.1	24.5	25.7	7.4
Skillfulness					
Assessing the patient's understanding of the cause of his or her illness^*^	21.8	62.9	74.6	82.3	60.5
Working effectively with a medical interpreter^*^	18.4	28.9	58.0	76.7	58.3
Negotiating with the patient about key aspects of the treatment plan^*^	17.8	27.0	45.3	63.1	45.3
Determining how a patient wants to be addressed and interacted with^*^	31.6	52.4	66.1	73.3	41.7
Taking a social history^*^	25.1	59.0	65.9	69.3	44.2
Identifying whether a patient is mistrustful of the healthcare system or physicians^*^	21.7	33.3	47.0	44.8	23.1
Counseling patients about their use of complementary or alternative medicine^*^	9.7	20.5	24.7	29.9	20.2
Identifying religious beliefs that might affect clinical care^*^	13.4	26.5	29.7	30.4	17.0
Identifying cultural (nonreligious) customs that might affect clinical care^*^	19.3	27.2	31.0	34.3	15.0
Identifying how well a patient can read or write English^***^	28.8	27.9	31.9	34.1	5.3

^*^ *p*<0.001, ^**^p=0.010, ^***^p=0.107—between year 1 and 4.

Statistically significant differences (*p*<0.001) between year 1 and 4 students were found for all but one preparedness item (caring for transgender patients). The top three items that showed the greatest improvement were caring for patients with LEP (+30.5%), from cultures different from one's own (+26.8%), and members of racial/ethnic minorities (+22.1%). For the 10 skillfulness items, all but one (identifying how well a patient can read or write English) showed statistically significant differences (*p*=0.001) between year 1 and 4.

**FIG. 1.**
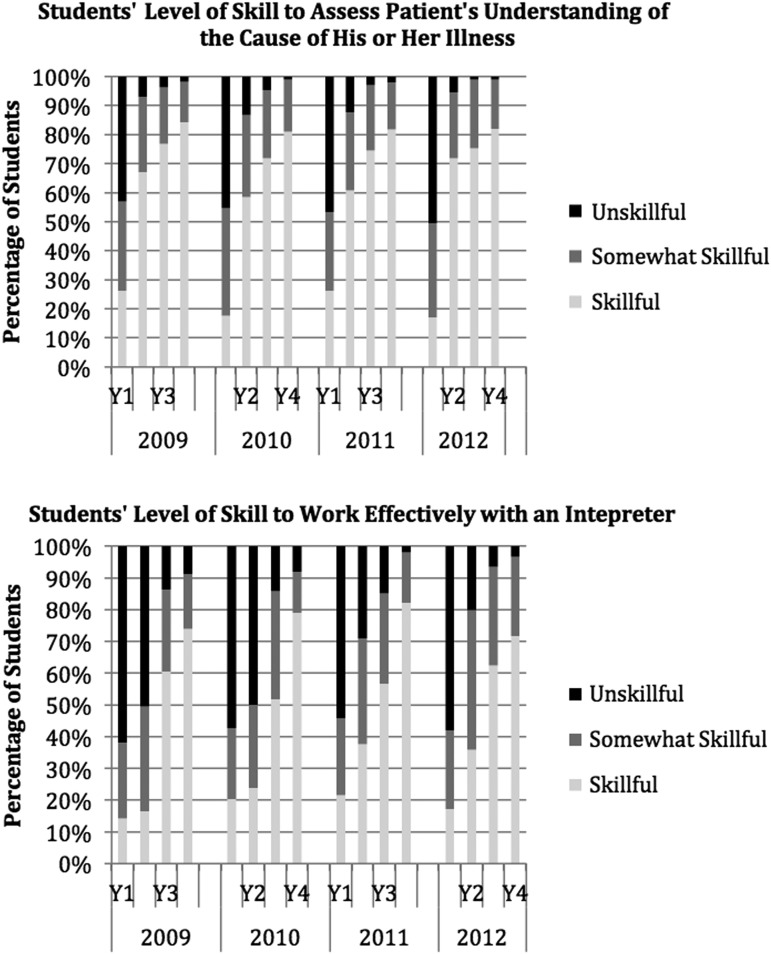
Skillfulness items showing greatest improvement across years of training, Harvard Medical School, 2009–2012.

**FIG. 2.**
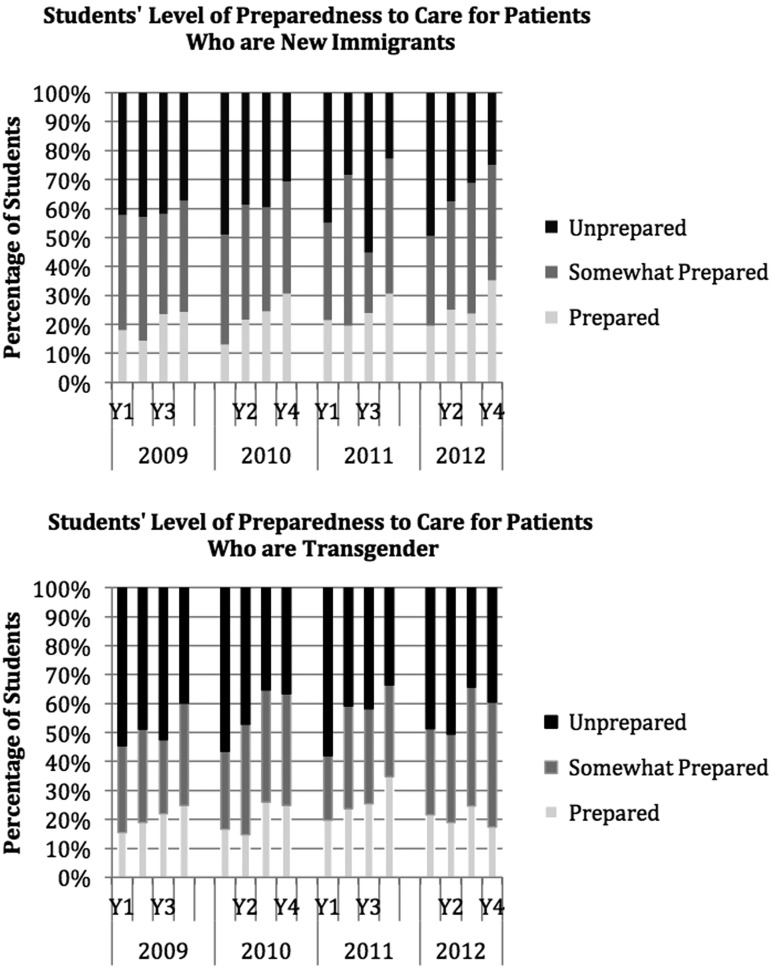
Preparedness items showing least improvement across years of training, Harvard Medical School, 2009–2012.

Figure 1 shows these findings in more detail graphically for two specific examples: assessing a patient's understanding of the cause of his or her illness and working effectively with an interpreter. Responses on the 5-point Likert scale are shown here as three outcome categories: unskillful (1 or 2), somewhat skillful (3), or skillful (4 or 5) and data for all 4 years are shown. For each of these skill items, there is a dramatic difference between two adjacent years. Figure 2 shows similar detailed examples for two preparedness items: caring for patients who are new immigrants and caring for patients who are transgender. These items show only small difference across the years of training.

### Learning environment

Of second-year students, 29.9% reported seeing greater than 20% racial and ethnic minority patients versus 54.2% and 52%, respectively, in years 3 and 4 (*p*<0.001). We excluded first-year students, who were just beginning their training, from these analyses. Similarly, 6.8% of second-year students reported seeing greater than 20% of patients with LEP patients versus 14.7% and 13.8%, respectively, in years 3 and 4 (*p*<0.001). Nearly all students in years 2–4 (95.5%) regarded seeing a diverse patient panel mix as moderately or very important. Table 3 shows areas that students considered as problems during their training. There was no strong consensus among fourth-year students on whether course directors and clinical faculty incorporated cross-cultural issues into their teaching, with the most common response being “Slightly agree” at ∼40.3% for course directors and 38.5% for clinical faculty.

**Table 3.** **Percentage of Students Who Believe the Following Issues Are a Problem (Small, Moderate, Big) Versus Not a Problem, Harvard Medical School, 2010–2012**

	% of respondents (*N*=1369)			
	Year 2	Year 3	Year 4	All respondents (year 2–4)
Lack of practical experience caring for diverse patient populations	77.5	74.8	68.8	73.5
Inadequate cross-cultural training during medical school	68.6	67.3	65.6	67.1
Absence of good role models or mentors for cross-cultural care among faculty	58.2	60.0	60.7	59.6
Dismissive attitudes about cross-cultural care among attending physicians	42.9	57.8	42.4	54.8
Dismissive attitudes about cross-cultural care among your fellow students	48.3	49.4	54.7	50.9

We excluded year 1 because data were not adequate due to early survey administration in year 1.

## Discussion

Our study utilized a validated survey of cross-cultural preparedness and skillfulness (the CCCS) to understand the perspectives of medical students at different points during their training to assess the effectiveness of the current curriculum. The CCCS is able to track students' perceived competency across a wide range of specific components of cross-cultural care over the 4 years of training. It points to areas where training appears either effective of deficient. It also helps identify at what point in the curriculum certain aspects of learning occur.

Most medical students even by their final year of training did not yet feel adequately prepared on most of the cross-cultural care preparedness items. This is not unexpected as medical students at the beginning of their fourth year still have relatively little experience caring for patients, and may rate themselves fairly low on any measure of “preparedness” to provide care (cross cultural or otherwise). An earlier survey of senior residents showed much higher rates of cross-cultural preparedness.^6^ The skills measures showed an overall higher competency by the fourth year, with particularly large gains for “exploring the patient's understanding (of the illness)” and “working effectively with a medical interpreter” (Fig. 1). These aspects of cross-cultural care are explicitly emphasized in the first- and second-year curriculum, respectively, corresponding to where the greatest increase in perceived skill occurred. However, less than 50% of fourth-year students felt adequately skilled on five of the 10 skills items. The lack of preparedness and skills among senior medical students is concerning, especially given the strong emphasis HMS places on cross-cultural care.

Regarding specific survey results, items for which we saw little improvement (e.g., caring for patients who are transgender or new immigrants) reflect areas in need of more explicit and targeted training (Fig. 2). For other preparedness items (caring for racial/ethnic minority and gay, lesbian, and bisexual patients), a large proportion of first-year students (around 50%) reported already feeling prepared, suggesting that social desirability bias may have played a role. We were surprised to find low skillfulness ratings even by the fourth year for counseling patients about their use of complementary or alternative medicine and identifying religious beliefs and cultural customs that might affect clinical care. These are core skills of cross-cultural care that students should learn before starting residency when dedicated teaching in this area is often lacking.

Regarding the learning climate for cross-cultural care, while almost all students felt it was important to see a diverse patient panel, nearly three-quarters reported lack of experience with diverse patients to be a problem in their education. Many reported problems with inadequate cross-cultural training (67%) and lack of role models (60%). More than half reported dismissive attitudes about cross-cultural care among attending physicians and fellow students. These data imply that the culture of medical education, even at an institution that focuses significant attention on these issues, still has not fully accepted and integrated cross-cultural care as a fundamental component of the curriculum, at least to the degree that students expect. They also suggest the existence of a strong hidden curriculum that may undermine current efforts in cross-cultural education. However, students' perceptions of the learning climate did not change substantially by class year, contrary to what is often assumed about the clinical training experience.

Our study has several limitations. Because we wished to preserve anonymity, we did not track individual students over time, which required us to perform cross-sectional analyses rather than use each student as his/her own control. Also, we assessed students at the beginning of the year, not the end, so any additional improvement through the fourth year remained unmeasured. We are unsure whether respondents reported more or less preparedness/skillfulness than nonrespondents. Also, the CCCS was validated with residents but not specifically with medical students. Finally, self-report is a rough measure of competency, although it has been shown to correlate fairly well.^6^ Future studies could track individual students over time, even into residency; do subanalyses by sociodemographic characteristics; and use objective measures of competency such as standardized patient assessments.

The CCCS is a helpful tool to assess medical students' perceived preparedness and skillfulness to care for culturally diverse patients. Both the preparedness and skillfulness measures showed changes across the 4 years and can serve as complementary and useful ways of tracking students' cultural competency. The CCCS can also serve as a curricular assessment tool and can help guide curricular change in areas of cross-cultural care where students are weak. Ultimately, we hope to use the results of this yearly survey both to assess the impact of the major curricular interventions that are now underway and to track individual students' perceived cultural competency longitudinally.

## Abbreviations Used

CCCS: Cross-Cultural Care Survey
HMS: Harvard Medical School
LEP: limited English proficiency
